# Supplementary material for: Solvent engineering for scalable fabrication of perovskite/silicon tandem solar cells in air
Source: Nat Commun. 2024 Jun 8;15:4907. doi: 10.1038/s41467-024-49351-5 (PMC11162483; doi:10.1038/s41467-024-49351-5)
Supplement: Supplementary file 1 — Supplementary Information [file 41467_2024_49351_MOESM1_ESM.pdf]

Supplementary Information for:

Solvent Engineering for Scalable Fabrication of Perovskite/Silicon Tandem Solar Cells in Air

Xuntian Zheng[1†], Wenchi Kong[1†], Jin Wen[1†], Jiajia Hong[1], Haowen Luo[1], Rui Xia[2], Zilong Huang[1], Xin Luo[1], Zhou Liu[1], Hongjiang Li[2], Hongfei Sun[1], Yurui Wang[1], Chenshuaiyu Liu[1], Pu Wu[1], Han Gao[1], Manya Li[1], Anh Dinh Bui[3], Yi Mo[2], Xueling Zhang[2], Guangtao Yang[2], Yifeng Chen[2], Zhiqiang Feng[2], Hieu T. Nguyen[3], Renxing Lin[1], Ludong Li[1], Jifan Gao[2*], Hairen Tan[1*]*

[1]*National Laboratory of Solid State Microstructures, College of Engineering and Applied Sciences, Frontiers Science Center for Critical Earth Material Cycling, Nanjing University, Nanjing 210023, China.*

[2]*State Key Laboratory of PV Science and Technology, Trina Solar, ChangZhou 210031 China*

[3]*Research School of Electrical, Energy and Materials Engineering, College of Engineering and Computer Science, The Australian National University, Canberra, NSW, Australia.*

†These authors contributed equally to this work.

*Corresponding authors. E-mail: kongwenchi@nju.edu.cn; jifan.gao@trinasolar.com; hairentan@nju.edu.cn

Supplementary Note 1: PLQY measurement:

Excitation for the PL measurements was performed with a 365 nm laser through an optical fiber into an integrating sphere. The intensity of the laser was adjusted to a 1 Sun equivalent intensity by illuminating a 0.049 cm2 size perovskite solar cell under short-circuit and matching the current density to the J_{SC} under the solar simulator. (e.g. ~21.5 mA cm^{-2} at 100 mW cm^{-2} for a $FA_{0.8}Cs_{0.15}MA_{0.05}Pb(I_{0.82}Br_{0.18})_3$ perovskite cell). The quasi-Fermi level splitting (QFLS) at 1 sun can be measured by the PLQY. Both in the neat absorber layer or in individual components of the multilayer device stack. [1]

In this manuscript, comparing the QFLS in the absorber layer with and without the transport layers allows quantification of the nonradiative losses at the perovskite/CTL interfaces. Therefore, to disentangle the origin of nonradiative recombination losses in the devices we calculated the QFLS of the neat perovskite films, perovskite/CTL stacks using the following relation between the QFLS and the PLQY junctions.

$$QFLS = K_B T \, ln\left(PLQY \, \frac{J_{inj}}{J_{0,rad}}\right) \quad (1)$$

In Equation (1), kB is the Boltzmann constant. T=25°C. J_G is the generation current density, which is equal to the J_{SC} in almost all cases. $J_{0,\,rad}$ is the radiative recombination current density in thermal equilibrium or in the dark, which is obtained from the overlap of the blackbody spectrum (at 300 K).

$$J_{0,rad} = q \int EQE_{PV}(E)_{\varphi BB}(E)dE$$

Supplementary Note 2: Open-circuit voltage loss

The EQE_{EL} of the single-junction and tandem device were determined by measuring the emitted photons of the devices in all directions through an integrated sphere by using a calibrated spectrometer (QE Pro, Ocean Optics), under a constant current density provided by a Keithley 2400 source measure unit. The EQE_{EL} allows us to quantify the QFLS in each subcell for each injection current J_{inj} via

$$QFLS_{EL} = K_B T \, ln\left(EQE_{EL} \, \frac{J_{inj}}{J_{0,rad}}\right),$$

in which K_B is the Boltzmann constant. T=25°C. J_{inj} is the generation current density, which is equal to the J_{SC} in almost all cases. $J_{0,rad}$ is the radiative recombination current density in thermal equilibrium or in the dark, which is obtained from the overlap

of the blackbody spectrum (at 300 K). For a solar cell with a bandgap of 1.68 eV, the $V_{OC,limit}$ (same as $QFLS_{rad}$) is calculated to be 1400 mV.

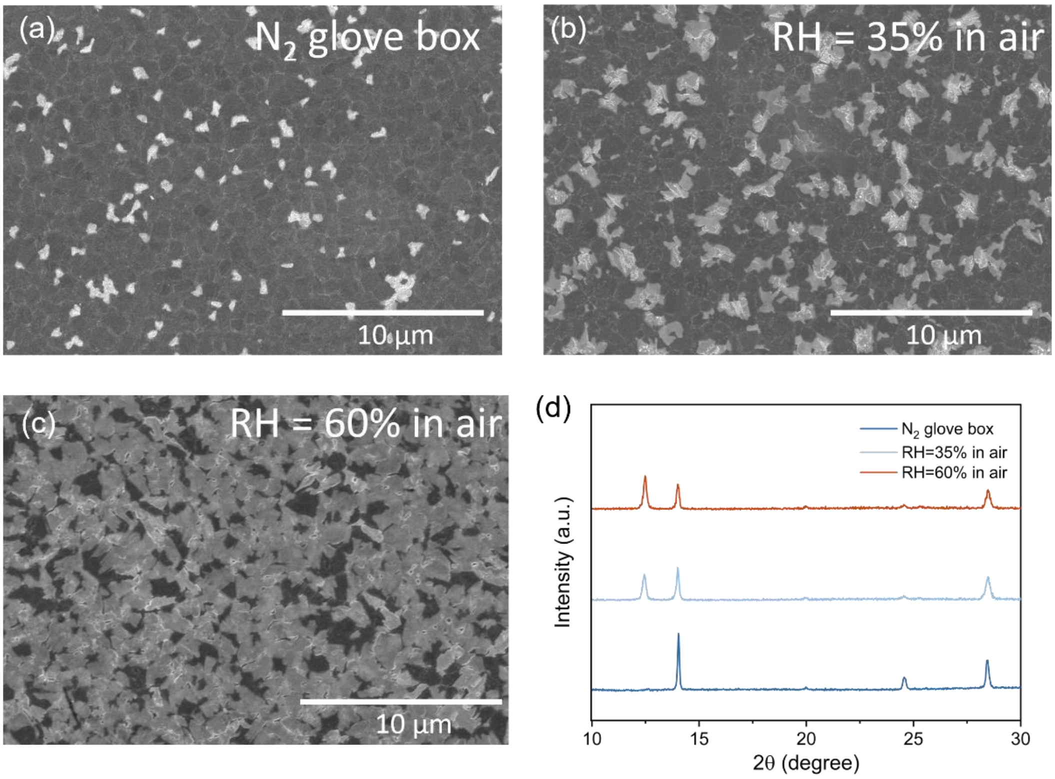

Supplementary Fig. 1 Top-view SEM images perovskite films fabricated in different condition **a**, in N_2 glove box. **b**, RH = 35% in air. **c**, RH = 60% in air. **d**, X-ray diffraction (XRD) patterns of perovskite films fabricated in different condition

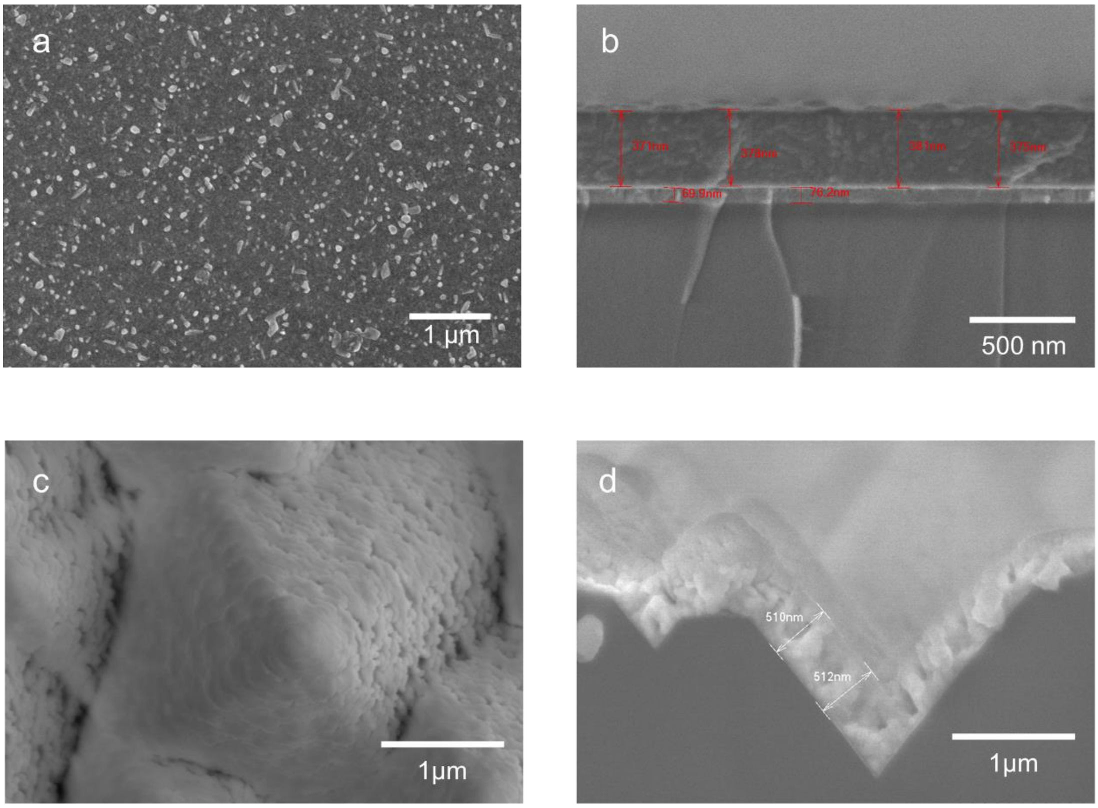

Supplementary Fig. 2 Top-view and cross-sectional SEM images of inorganic framework fabricated on glass **a, b,** and on textured silicon **c, d**.

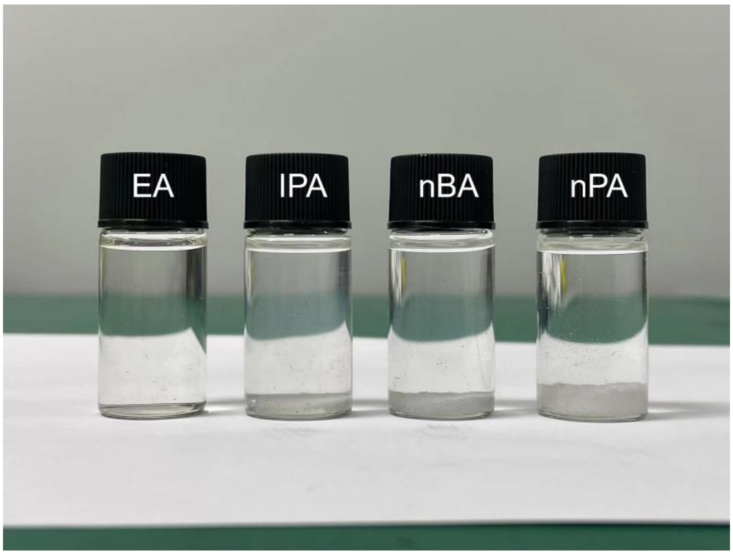

Supplementary Fig. 3 Images of different alcohols dissolving organic salts (FAI, FABr, MACl, MASCN).

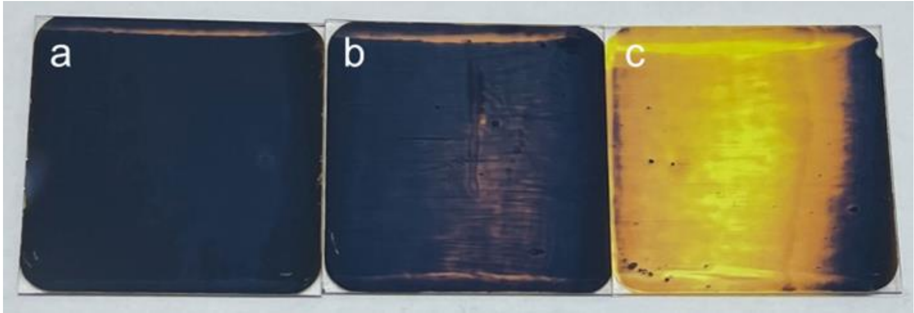

Supplementary Fig. 4 Images of perovskite films in different reaction stages fabricated by IPA. **a**, appropriate amount of organic salts. **b**, slightly excessive organic salts. **c**, excessive organic salts.

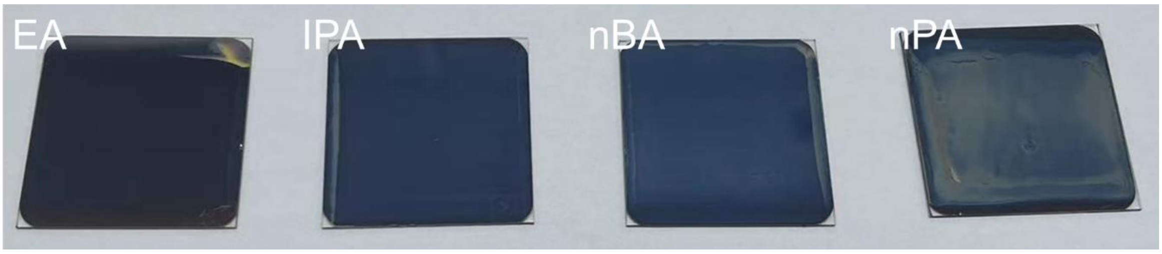

Supplementary Fig. 5 Images of final perovskite films fabricated by different alcohols.

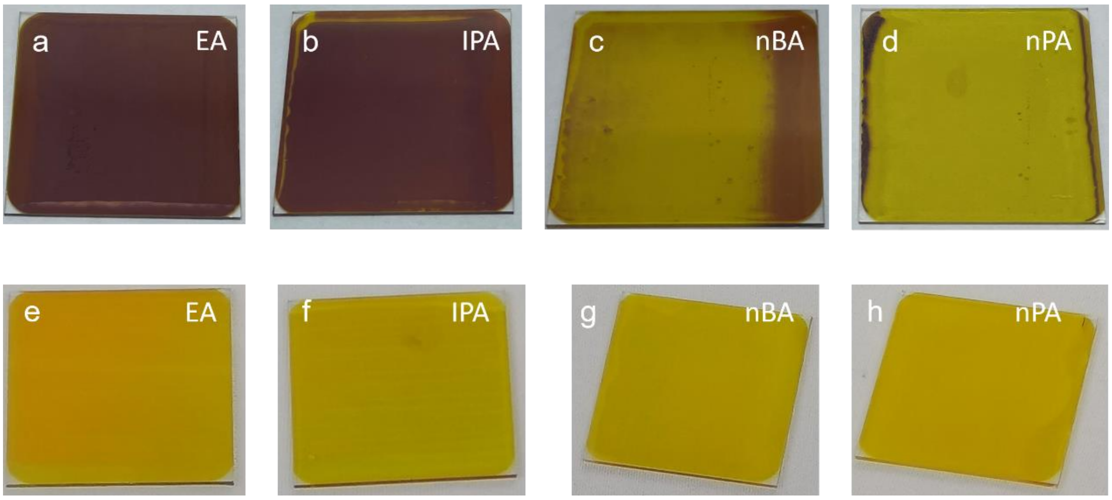

Supplementary Fig. 6 Images of films fabricated by various alcohols in different environments. **a, b, c** and **d** in air and **e, f, g** and **h** in N_2 environment.

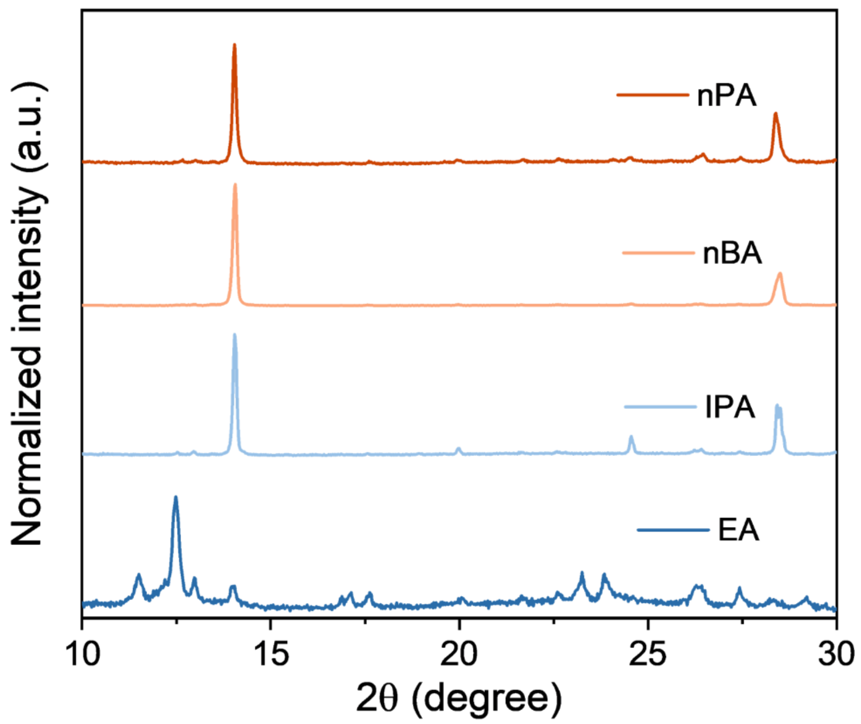

Supplementary Fig. 7 X-ray diffraction (XRD) patterns of perovskite films fabricated by various alcohols before annealing.

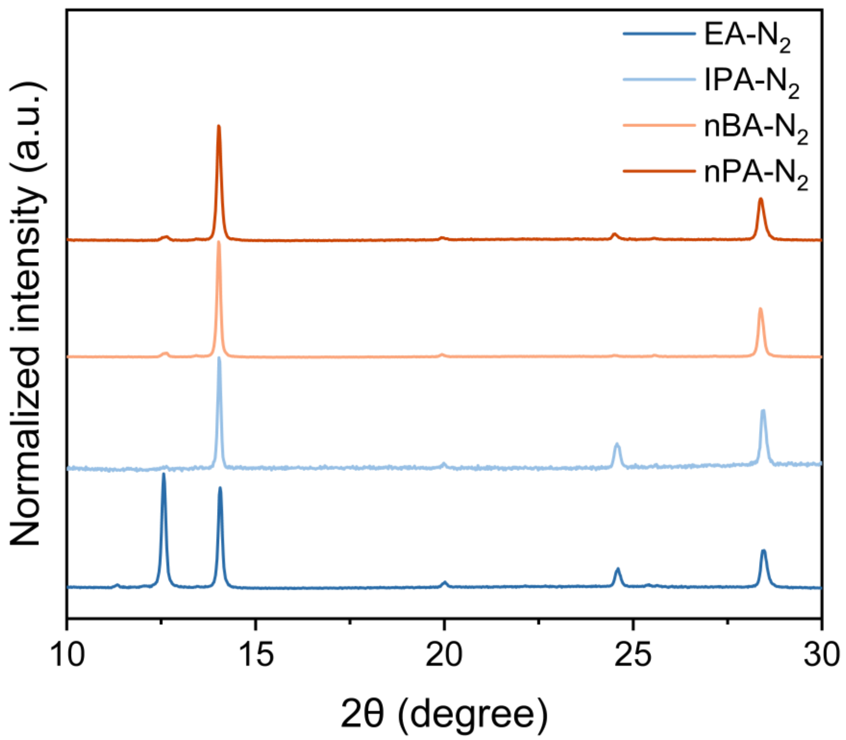

Supplementary Fig. 8 XRD patterns of perovskite films fabricated by different alcohols in N_2 environment.

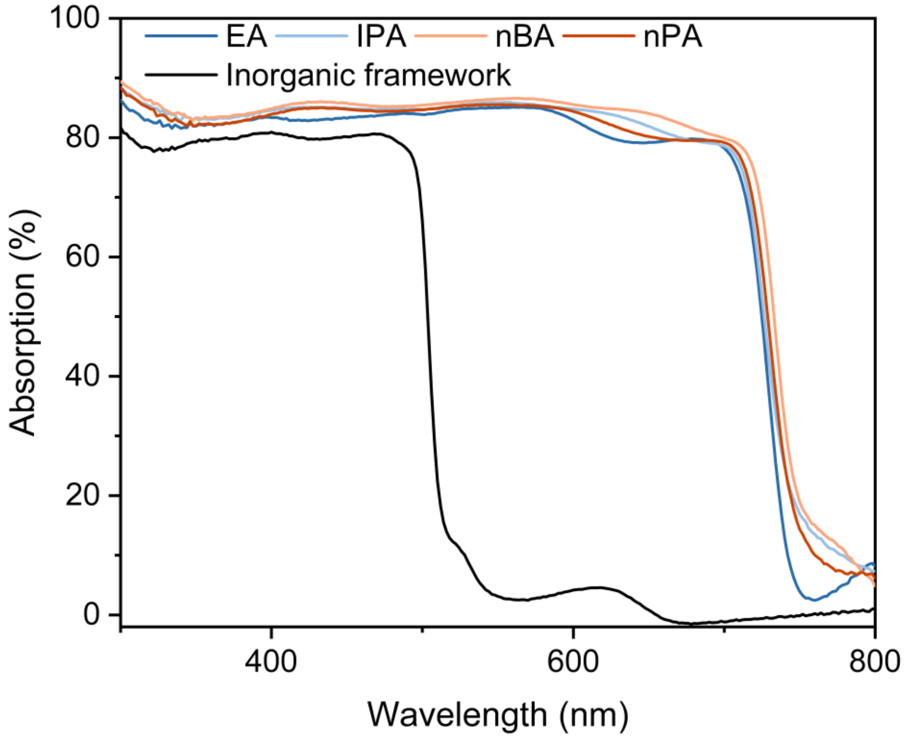

Supplementary Fig. 9 UV-vis spectra of perovskite films fabricated by various alcohols and inorganic framework.

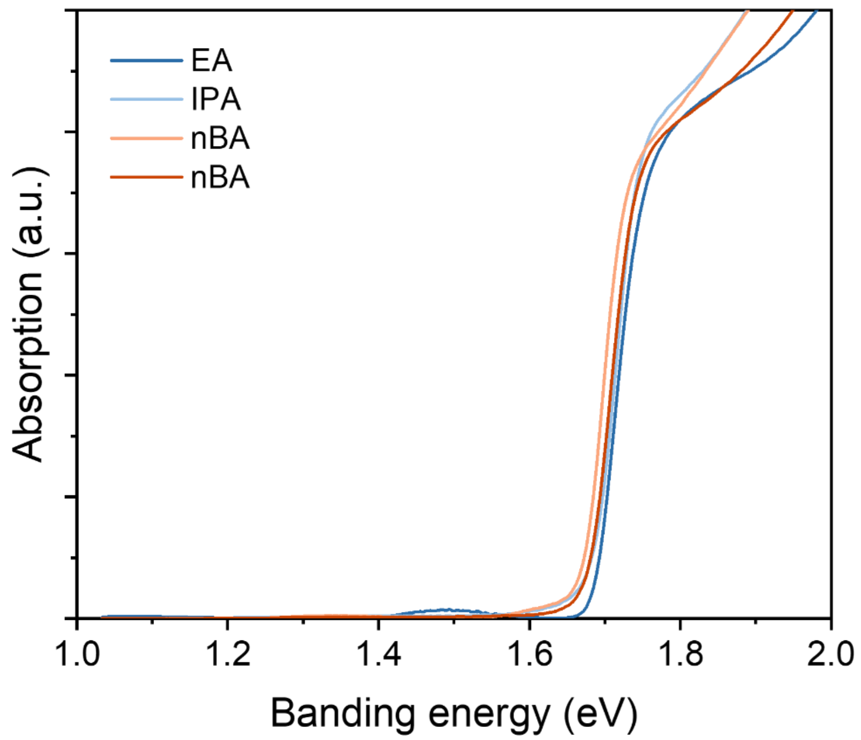

Supplementary Fig. 10 Tauc-plots of perovskite films fabricated by various alcohols.

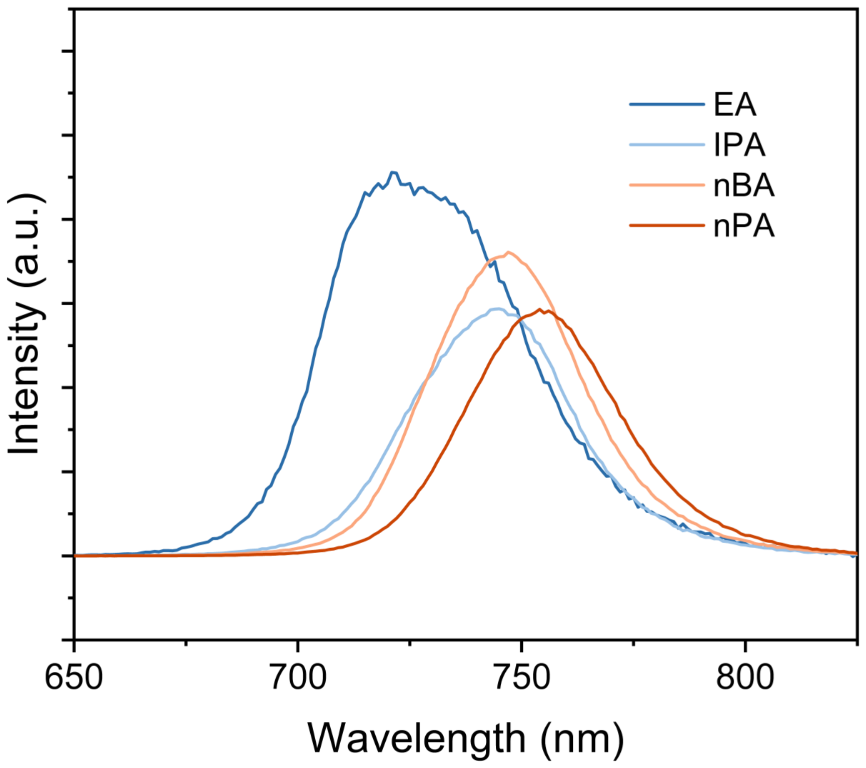

Supplementary Fig. 11 Steady-state PL spectra of perovskite films on glass with the emission from the perovskite film side.

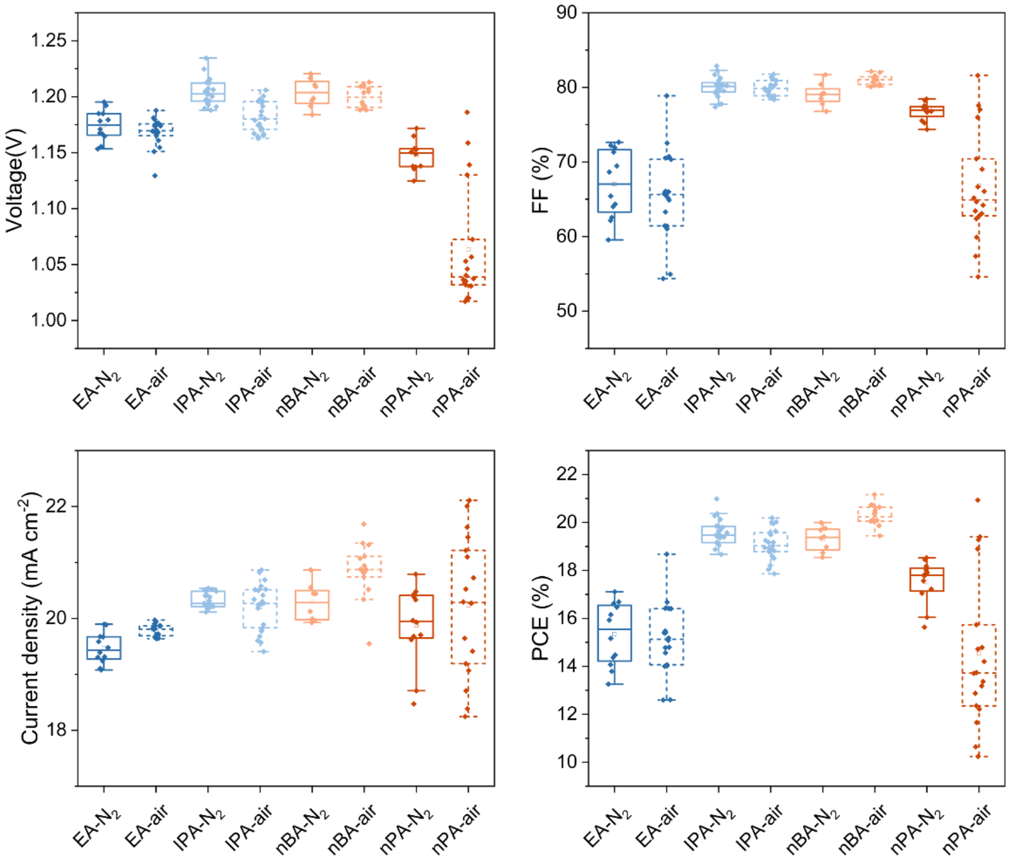

Supplementary Fig. 12 Photovoltaic parameter of devices fabricated by different alcohols in air and N_2 environment.

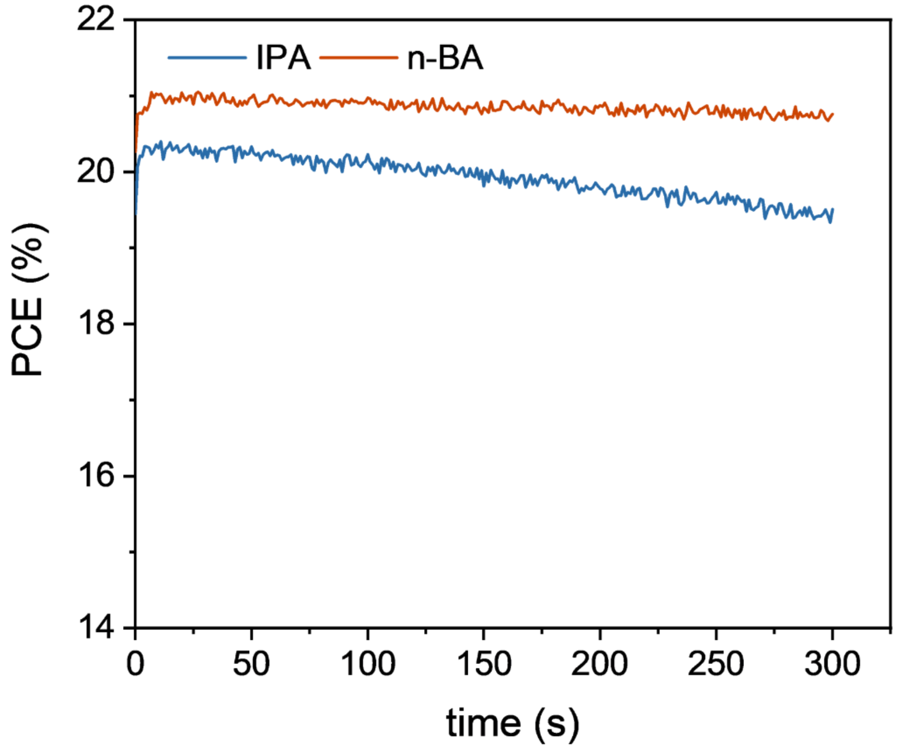

Supplementary Fig. 13 MPP tracking of the 1.68 eV-bandgap single junction.

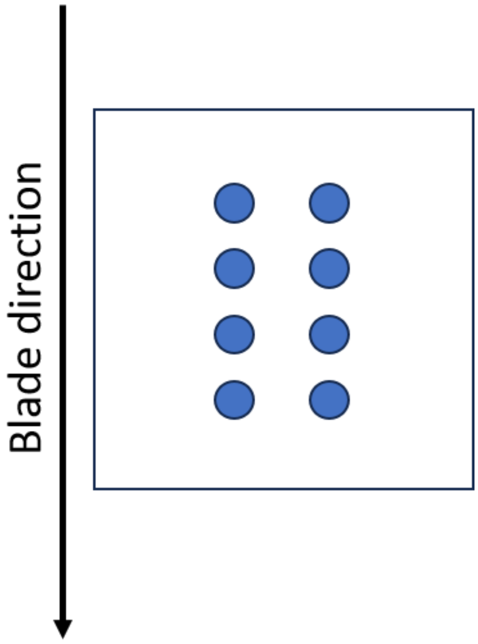

Supplementary Fig. 14 Schematic architecture of 8 separate 0.049 cm^2 perovskite solar cells.

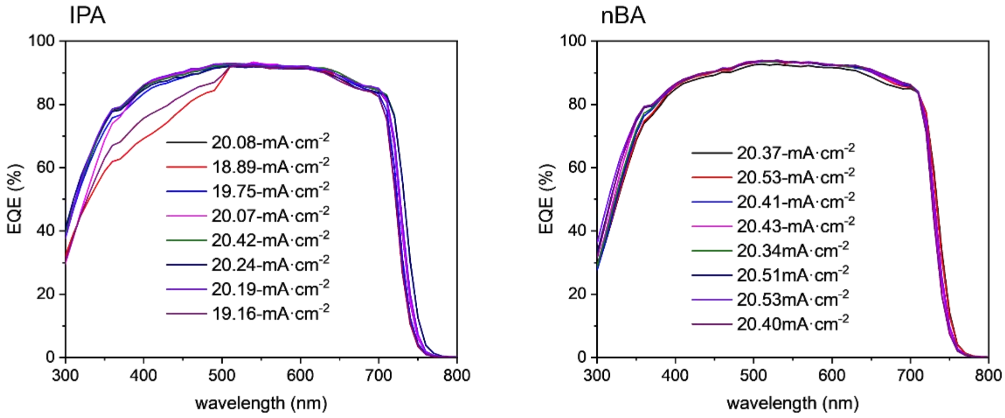

Supplementary Fig. 15 EQE spectra of 8 separate 0.049 cm^2 perovskite solar cells.

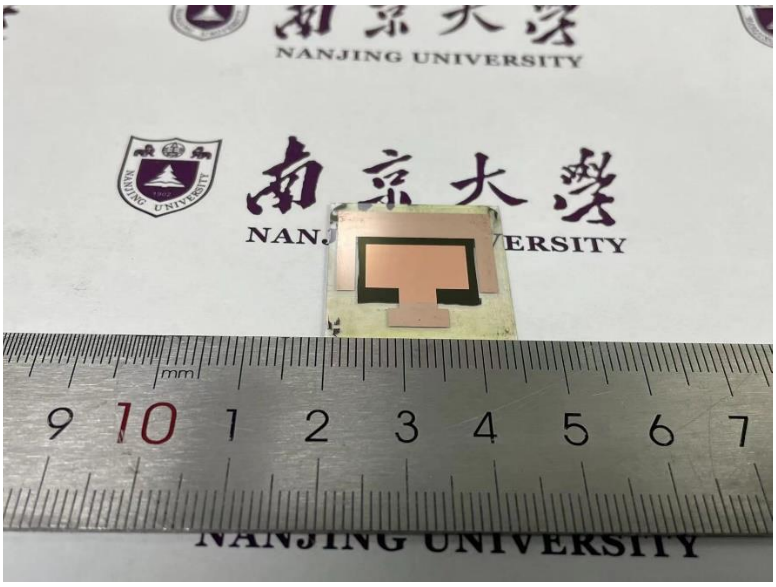

Supplementary Fig. 16 The digital photograph of 1.044 cm^2 device.

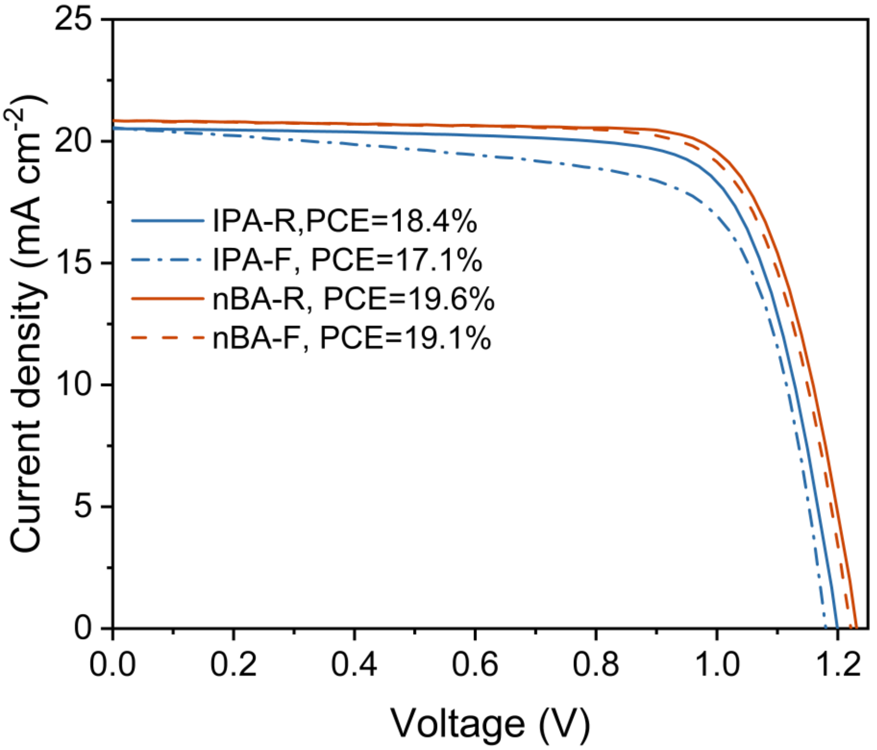

Supplementary Fig. 17 *J*–*V* curves of the champion devices (1.044 cm^2 aperture area).

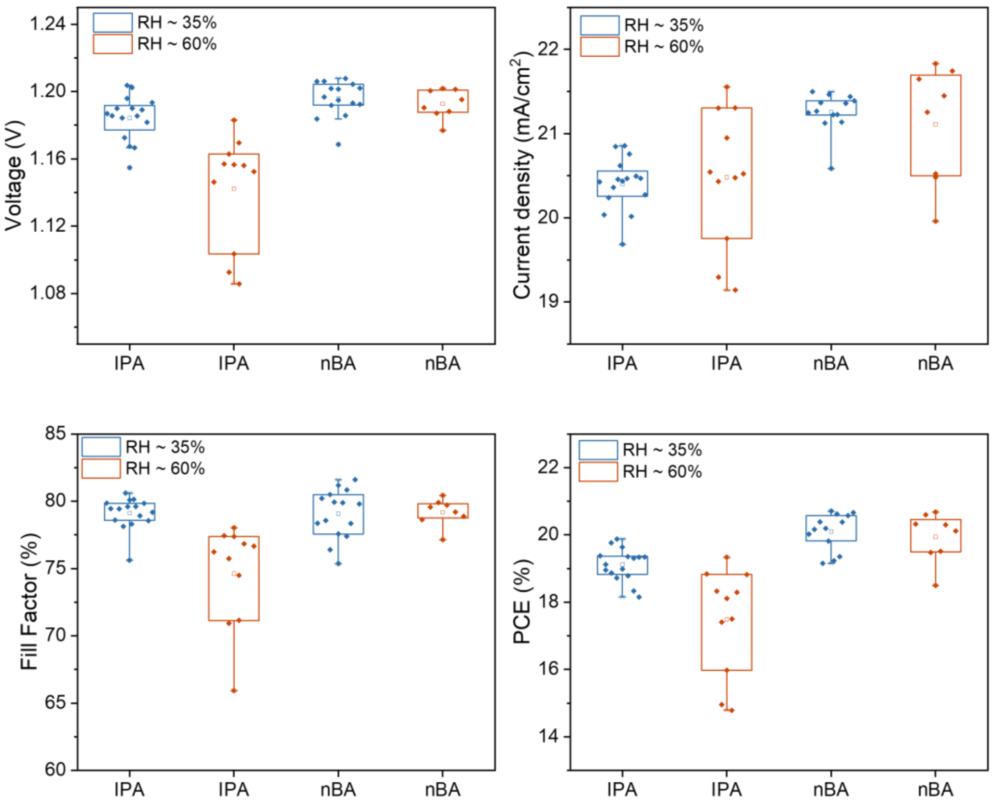

Supplementary Fig. 18 Photovoltaic parameter of IPA and nBA devices fabricated under different humidity.

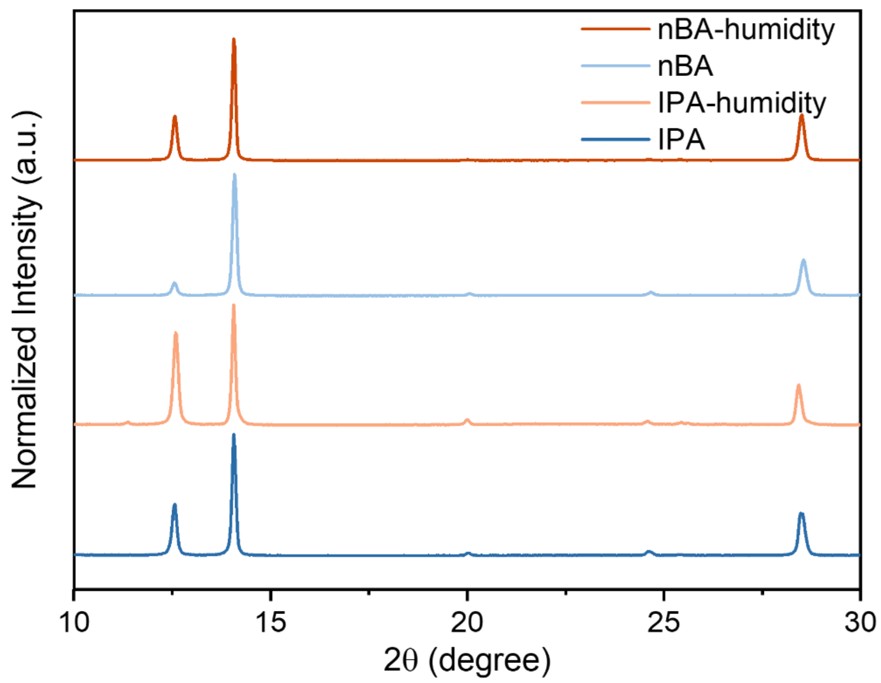

Supplementary Fig. 19 XRD patterns of IPA and nBA devices fabricated under different humidity.

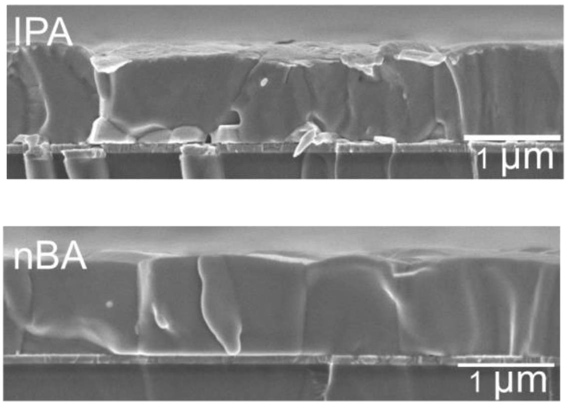

Supplementary Fig. 20 Cross-sectional SEM images of thicker perovskite films.

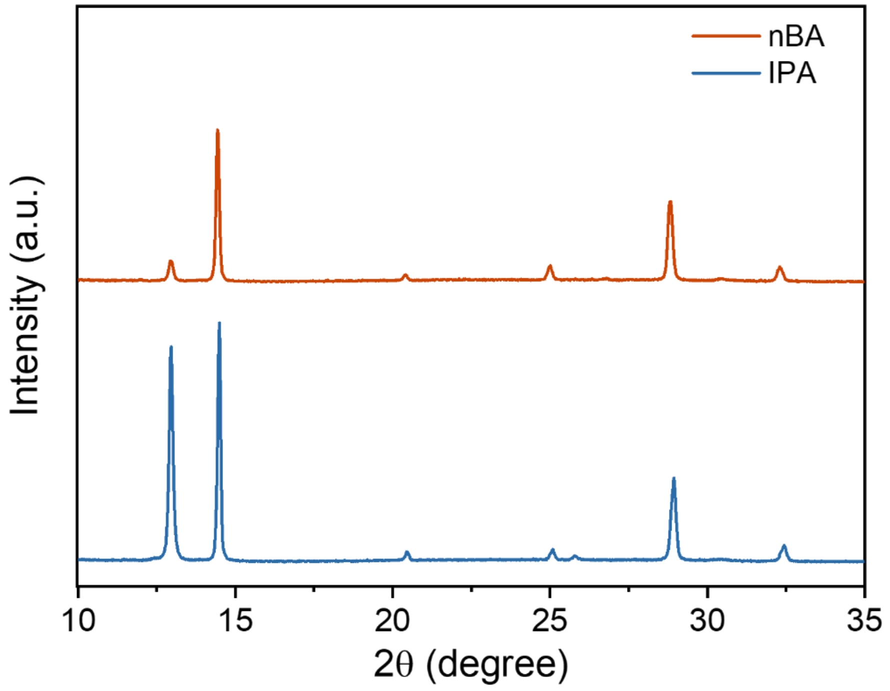

Supplementary Fig. 21 XRD patterns of thicker perovskite films.

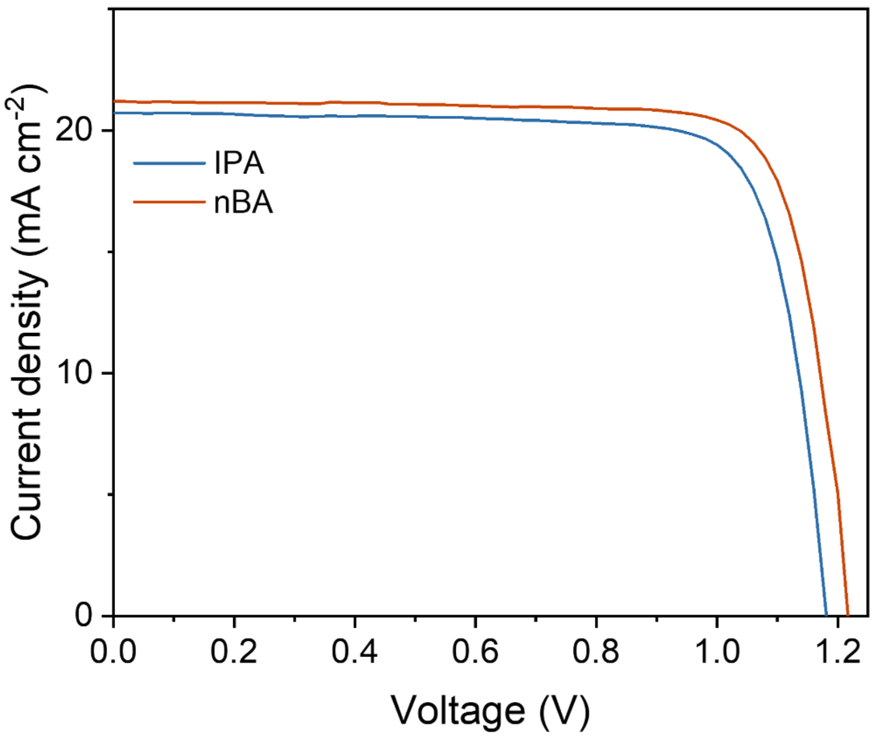

Supplementary Fig. 22 *J*–*V* curve of the champion device with thicker perovskite layer.

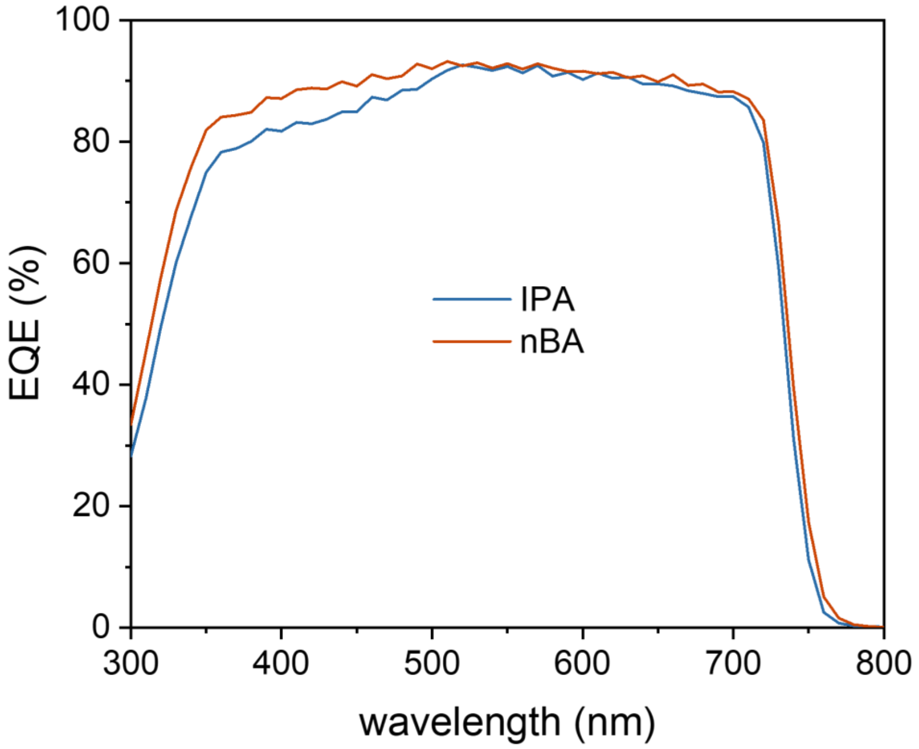

Supplementary Fig. 23 EQE curves of the champion devices with thicker perovskite layer.

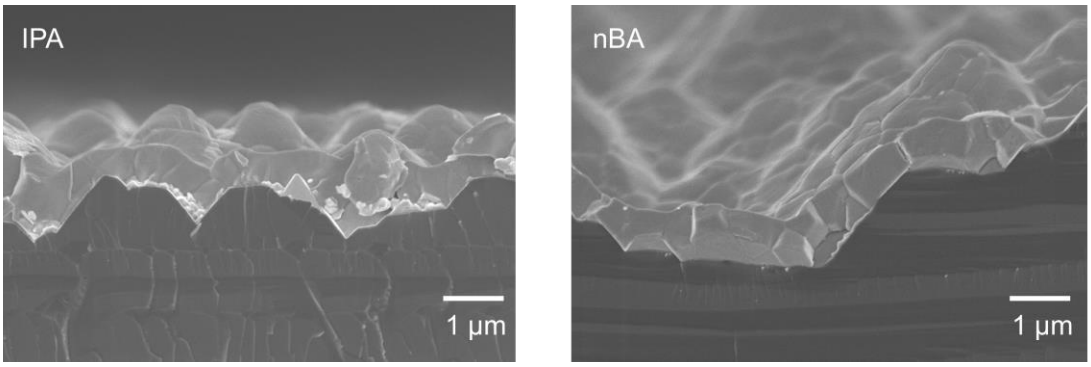

Supplementary Fig. 24 Cross-sectional SEM images of perovskite films on textured silicon heterojunction (SHJ).

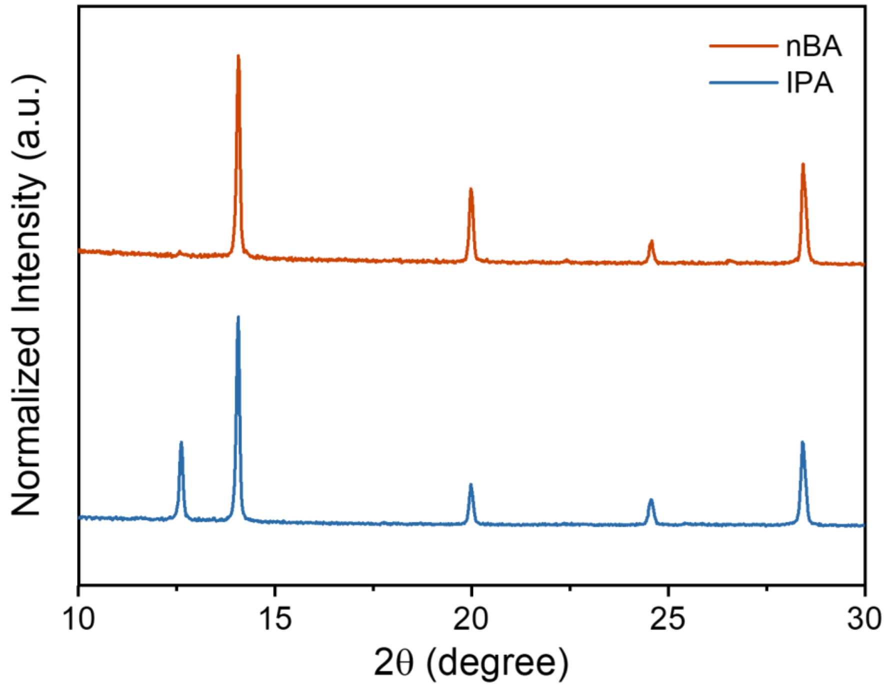

Supplementary Fig. 25 XRD patterns of perovskite films on textured SHJ.

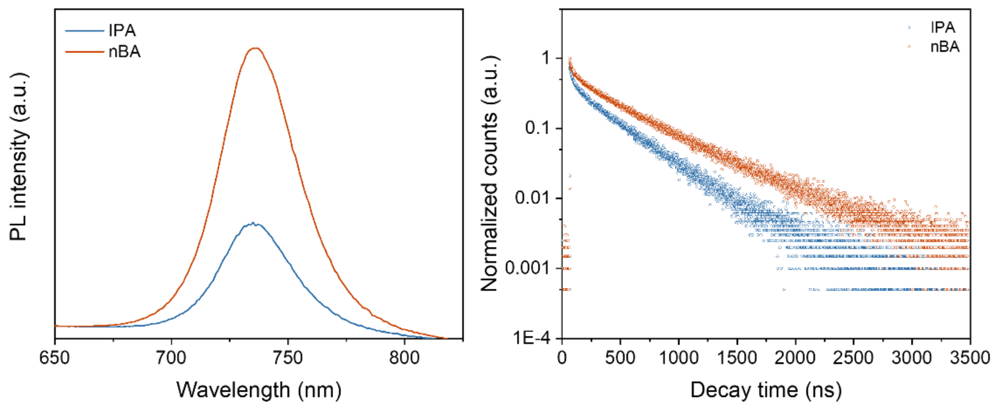

Supplementary Fig. 26 Steady-state PL spectra, and time-resolved PL transients of perovskite films on textured SHJ.

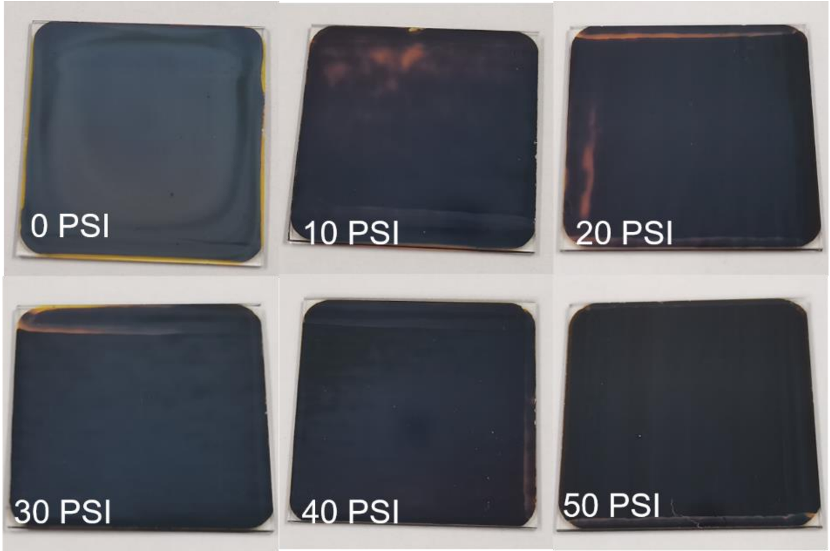

Supplementary Fig. 27 Images of IPA films fabricated using different quenching gas pressure.

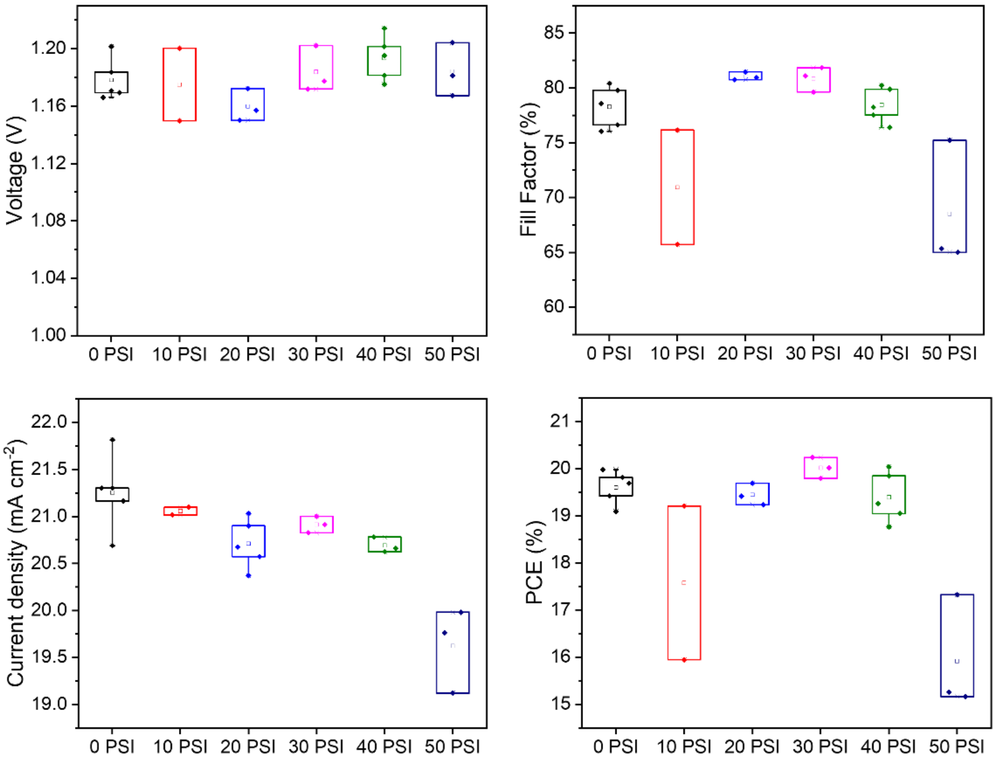

Supplementary Fig. 28 Photovoltaic parameters of IPA films fabricated using different quenching gas pressure.

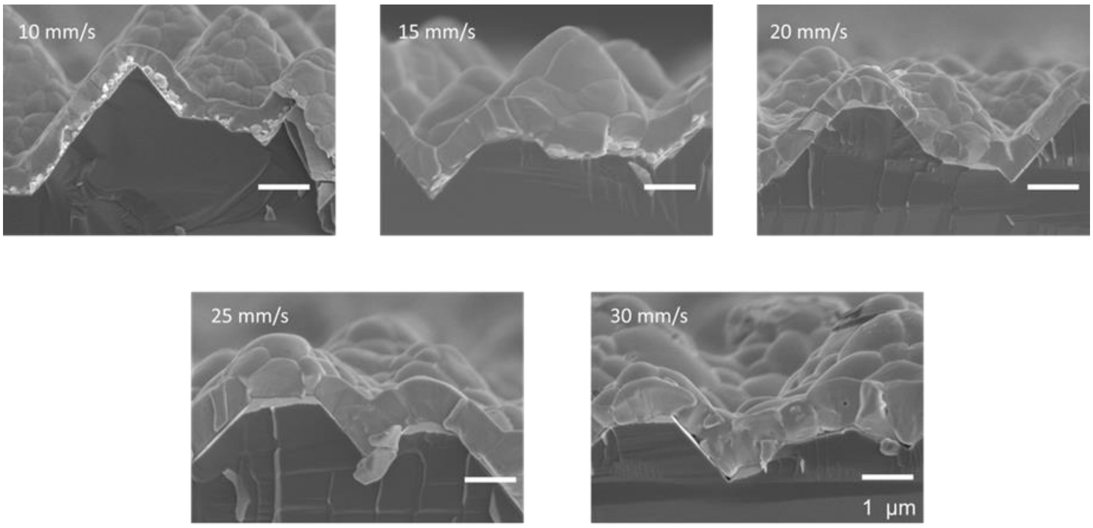

Supplementary Fig. 29 Cross-sectional SEM images of perovskite films fabricated on textured silicon with different blade-coating rate.

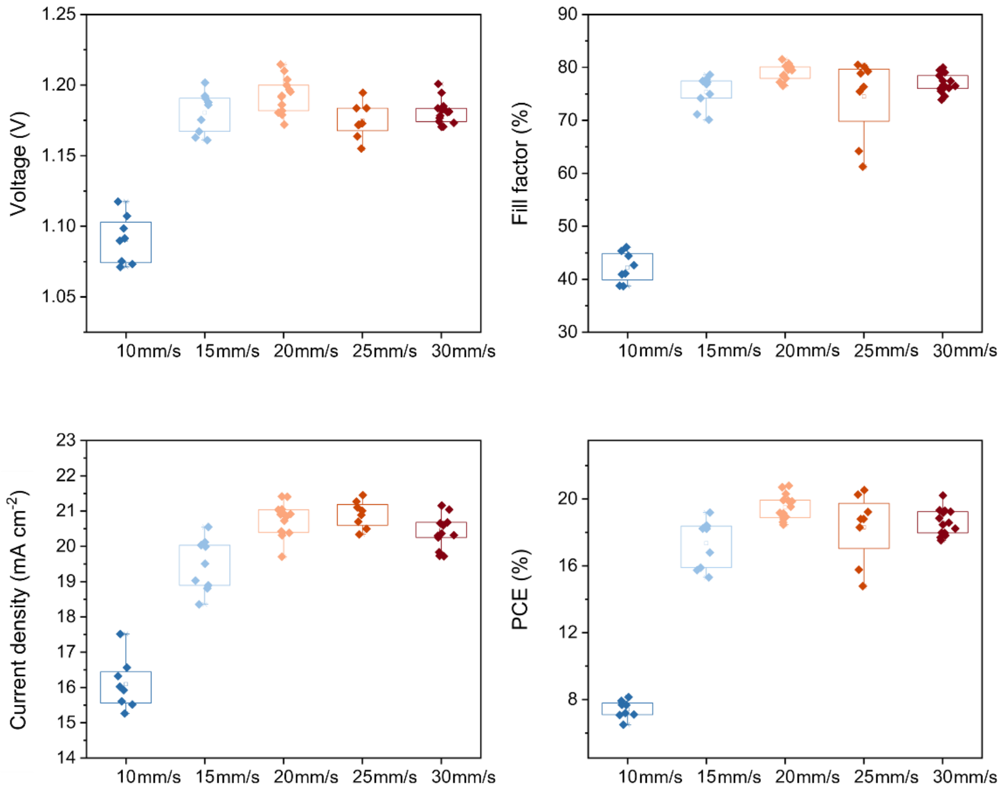

Supplementary Fig. 30 Photovoltaic parameters of IPA devices fabricated using different blade-coating rate.

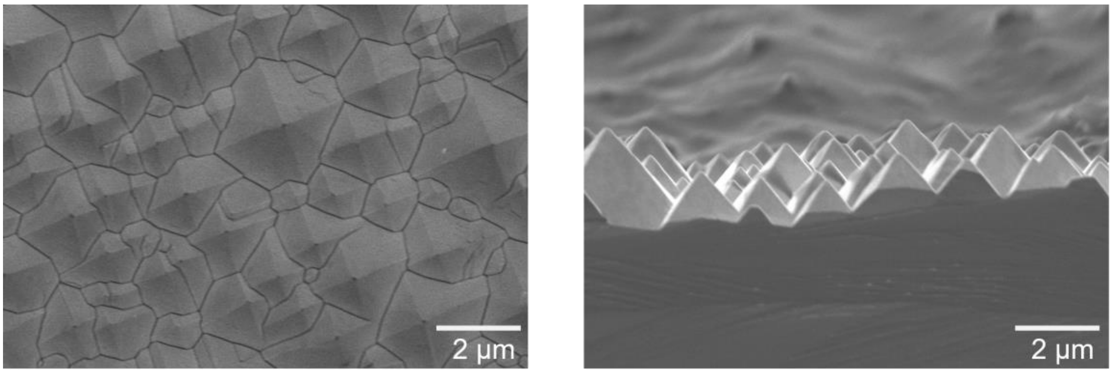

Supplementary Fig. 31 Top-view (right) and cross-sectional (left) SEM images of textured silicon heterojunction.

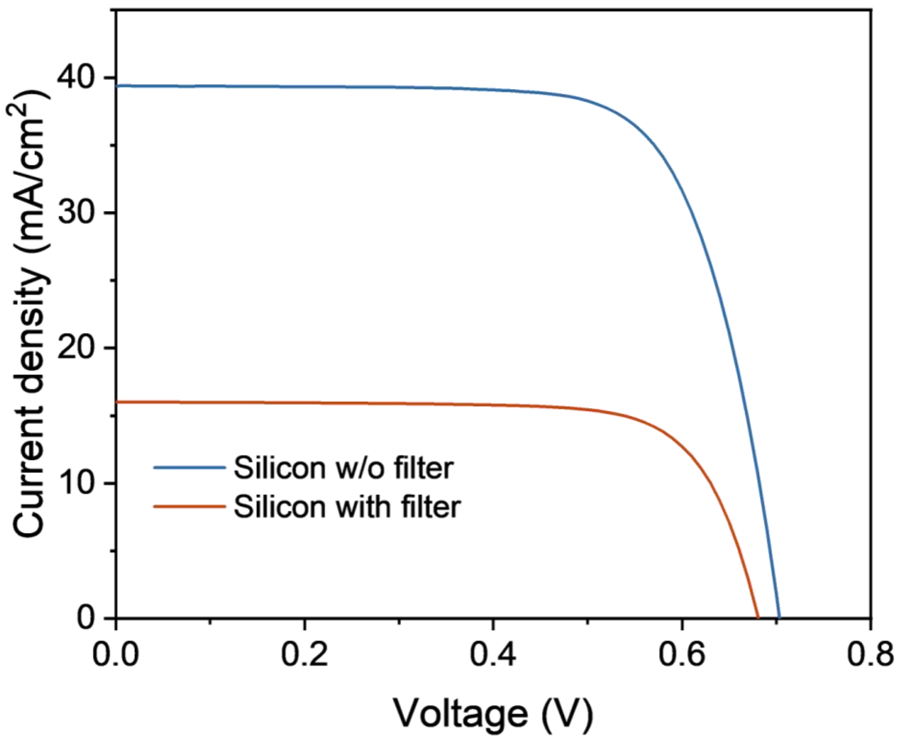

Supplementary Fig. 32 *J–V* curve of the textured silicon heterojunction with and without the perovskite filter.

Fraunhofer ISE CalLab PV Cells

Heidenhofstr.2

79110 Freiburg

Fraunhofer ISE

CalLab PV Cells

Werkskalibrierschein
Proprietary calibration report

10002070TSE0523

Gegenstand<br>*Object*	**monofacial multi-junction solar cell**
Hersteller<br>*Manufacturer*	
Typ<br>*Type*	**PSC/Si**
Fabrikat/Serien-Nr.<br>*Serial number*	**TSE002 / 2**
Auftraggeber<br>*Customer*	**NanJing University/ Trina solar Co., Ltd.**<br>**No.2 Tianhe Road,Trina PV Industrial Park ,Xinbei district**<br>**213032 Changzhou, Jiangsu province**<br>**China**
Auftragsnummer<br>*Order No.*	**070TSE0523**
Anzahl der Seiten<br>*Number of pages*	**6**
Datum der Kalibrierung<br>*Date of calibration*	**31.05.2023**

Kalibrierscheine ohne Unterschrift haben keine Gültigkeit. *Calibration certificates without signature are not valid.*

Datum<br>*Date*	Leiter des Kalibrierlaboratoriums<br>*Head of the calibration laboratory*	Bearbeiter<br>*Person in charge*
13.06.2023	*Jochen Hohl-Ebinger*	*Astrid Semeraro*

Fraunhofer ISE

CalLab PV Cells

10002070TSE0523

1. Beschreibung des Kalibriergegenstandes

Description of the calibrated object

Das Messobjekt ist eine Tandem-Solarzelle . Typ: PSC/Si.
The device under test is a PSC/Si tandem solar cell.

2. Messverfahren

Measurement procedure

Die Kalibrierung des Kalibrierobjektes wird gemäß /1/ mit einem Zweilampen-DC-Sonnensimulator durchgeführt. Die Einstrahlung wird mit Hilfe einer Monitorzelle während der gesamten Messdauer aufgenommen und deren Schwankungen bezüglich der Messung linear korrigiert. Die Divergenz der Randstrahlen ist < 5°. Die Solarzelle wird auf einem Vakuumprobentisch thermisch stabilisiert.
The calibration of the test sample was performed at Standard Testing Conditions (STC) with a dual light steady-state solar simulator according to /1/. The irradiance is controlled with a monitor cell during the measurement in order to correct fluctuations linear. The divergence of the peripheral beams is < 5°. The solar cell is kept at a constant

Rückführung der Referenzsolarzellen/*Traceability of the reference solar cells* :

Identitäts-Nr. / *Identity-Nr.* :	Kalibrierschein-Nr./ *Certificate-Nr.* :	Rückführung/ *Traceability* :
ISE020008	47056-PTB-20	PTB
ISE021030	47077-PTB-21	PTB

Die Korrektur der spektralen Fehlanpassung (Mismatch), die durch die Abweichung der spektralen Verteilung des Sonnen Simulators vom Standard-Spektrum AM1.5G /3/ in Kombination mit den verschiedenen spektralen Empfindlichkeiten von Referenzzelle und Messobjekt entsteht /4/, wurde durch eine erweiterte Mismatchberechnung /4/ - wie in /2/ beschrieben - korrigiert.
Dazu wurde die spektrale Verteilung der Bestrahlung (Sonnensimulator) mit einem Spektralradiometer und die spektrale Empfindlichkeit des Messobjektes mit einem gitterbasierten Messplatz /5/ gemessen.
The spectral mismatch - caused by the deviation of the simulator spectrum from the standard spectrum AM1.5G /3/ in combination with the difference between the spectral response of the reference cell and that of the device under test (DUT) – is calculated by a generalized mismatch correction /4/ as described in /2/.
For the spectral mismatch correction the spectral distribution of the solar simulator is measured with a spectroradiometer, the spectral response of the DUT is measured with a grating-based setup according to /5/ .

Kalibrierzeichen Spektrale Empfindlichkeit / *Calibration mark spectral response* :

Der P_{MPP} wurde durch MPP-Tracking über 300s bestimmt. Der angegebene P_{MPP} ist der Mittelwert (Zeitbereich s.u.) dieser stabilisierten Messung. Anschließend wurde die IV-Kennlinie in zwei Richtungen (V_{OC} -> I_{SC} und I_{SC} -> V_{OC}) aufgenommen.
The P_{MPP} was determined by MPP-Tracking for 300s. The reported P_{MPP} represents the average value of the range (time range s.b.) of this stabilized measurement. Afterwards, the IV-curve was determined with a scan in both directions (V_{OC} -> I_{SC} and I_{SC} -> V_{OC}).

Zeitbereich / *Time range* : 601 - 900 s

Fraunhofer ISE

CalLab PV Cells

Die Rückführung der Spektralmessung auf SI-Einheiten erfolgte über den Vergleich mit einer Standardlampe.
The traceability of the measurement of the spectral distribution to SI-Units is achieved using a standard lamp for the calibration of the spectroradiometer.

Identitäts-Nr. / *Identity-Nr.* :	Kalibrierschein-Nr./ *Certificate-Nr.* :	Rückführung/ *Traceability* :
BN-9101-451	40006-20-PTB	PTB

3. Messbedingungen

Measurement conditions

Standardtestbedingungen (STC) / *Standard Testing Conditions (STC)* :

Absolute Bestrahlungsstärke / *Total irradiance* :	1000	W/m²
Nominalwert der Temperatur des Messobjektes / *Nominal Value of Temperature of the DUT* :	25	°C
Spektrale Bestrahlungsstärke / *Spectral irradiance distribution* :	AM1.5G Ed.4 (2019)	

Die Messung der IV-Kennlinie (Strom-Spannungs-Kennlinie) des Messobjektes erfolgt mit Hilfe eines Vierquadranten-Netzteiles und eines Kalibrierwiderstandes. Die Temperatur der Solarzelle wird mit einem Tastsensor ermittelt und auf (25±0,5)°C eingestellt.
The measurement of the IV-curve is performed with a 4-quadrant power amplifier and a calibration resistor. The temperature of the solar cell is determined by a sensor and adjusted to (25±0.5)°C.

4. Messergebnis

Measurement results

Fläche / *Area* (da)[1]: = (1.0350 ± 0.0064) cm²

[1] *: (t) = total area, (ap) = aperture area, (da) = designated illumination area /6/*

Kennlinienparameter des Messobjektes unter Standardtestbedingungen (STC) / *IV-curve parameter under Standard Testing Conditions (STC)* :

		Vorwärtsrichtung / *forwards scan direction*	Rückwärtsrichtung / *reverse scan direction*	steady state MPP
V_{oc}	=	(1834 ± 18) mV	(1845 ± 19) mV	
I_{SC} (Ed.2 - 2008)	=	(20.81 ± 0.40) mA	(20.85 ± 0.40) mA	
I_{MPP}	=	18.60 mA	19.03 mA	(19.07 ± 0.58) mA
V_{MPP}	=	1502 mV	1560 mV	(1556 ± 35) mV
P_{MPP}	=	27.9 mW	29.7 mW	(29.7 ± 1.2) mW
FF	=	73.2 %	77.2 %	
η	=			(28.7 ± 1.2) %

Angegeben ist jeweils die erweiterte Messunsicherheit, die sich aus der Standardmessunsicherheit durch Multiplikation mit dem Faktor *k*=2 ergibt. Sie wurde gemäß dem "Guide to the expression of Uncertainty in Measurement" ermittelt. Sie entspricht bei einer Normalverteilung der Abweichungen vom Messwert einer Überdeckungswahrscheinlichkeit von 95%.
The expanded measurement uncertainty resulting from the standard measurement uncertainty multiplied with a factor k=2 is specified. The calculation was carried out according to the "Guide to the expression of Uncertainty in Measurement". The value corresponds to a Gaussian distribution denoting the deviations of the measurement value within a probability of 95%.

5. Zusatzinformationen

Additional information

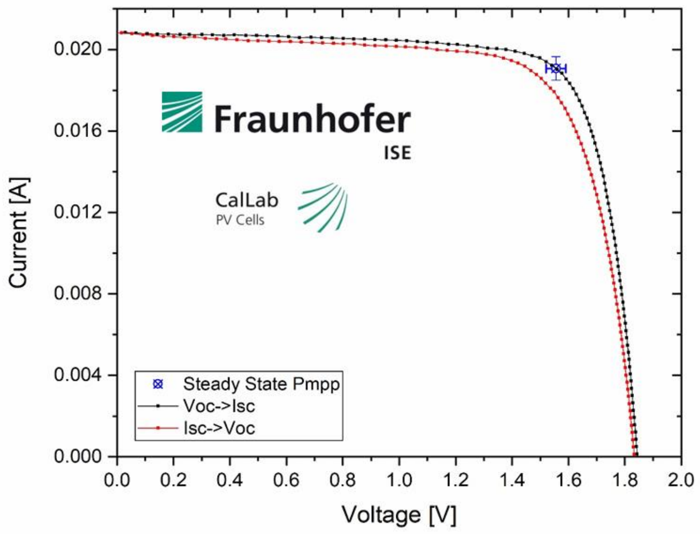

Fraunhofer ISE

CalLab PV Cells

10002070TSE0523

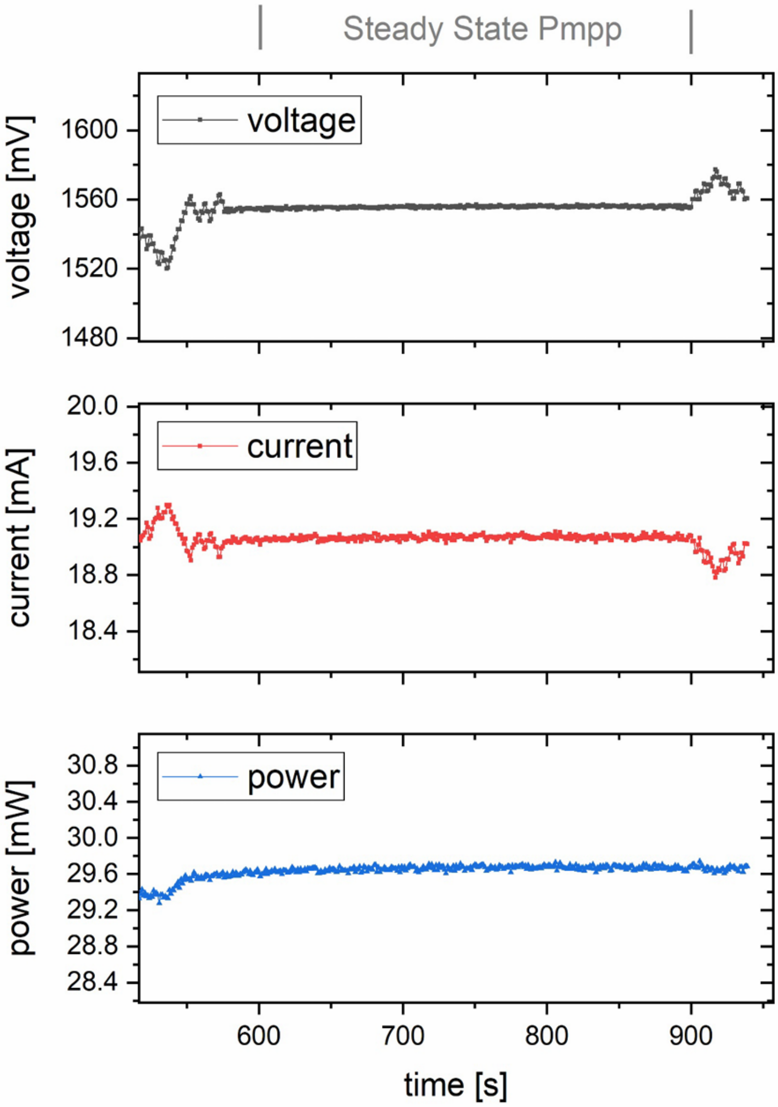

Fraunhofer ISE

CalLab PV Cells

10002070TSE0523

6.Literatur

Literature

/1/ IEC 60904-1-1:2017, *Photovoltaic devices - Part 1-1: Measurement of current-voltage characteristics of multi-junction photovoltaic (PV) devices*

/2/ M. Meusel, R. Adelhelm, F. Dimroth, A.W. Bett, W. Warta Spectral Mismatch Correction and Spectrometric Characterization of Monolithic III–V Multi-junction Solar Cells Prog. Photovolt: Res. Appl. 10 (2002) p. 243–255

/3/ IEC 60904-3-Ed.4:2019, *Photovoltaic devices - Part 3: Measurement principles for terrestrial photovoltaic (PV) solar devices with reference spectral irradiance data*

/4/ IEC 60904-7-Ed.4:2019, *Photovoltaic devices - Part 7: Computation of the spectral mismatch error introduced in the testing of a photovoltaic device*

/5/ IEC 60904-8-1:2017, *Photovoltaic devices - Part 8-1: Measurement of the spectral responsivity of multi-junction photovoltaic (PV) devices*

/6/ M.A. Green, K. Emery, Y. Hishikawa, W. Warta, and E. D. Dunlop, *Solar cell efficiency tables (version 39)*. Progress in Photovoltaics: Research and Applications, 2012. 20: p. 12-20.

Hinweis: Es ist nicht gestattet, ohne die schriftliche Genehmigung des ISE CalLab PV Cells den Werkskalibrierschein auszugsweise zu vervielfältigen.
Note: *This proprietary calibration report may not be reproduced other than in full. Extracts may be taken only by the written permission of ISE CalLab PV Cells.*

Ende des Kalibrierscheins / End of certificate

Supplementary Fig. 33 Certification reports from Fraunhofer ISE for a monolithic perovskite silicon tandem solar cell. This certificate is with written permission from Fraunhofer ISE.

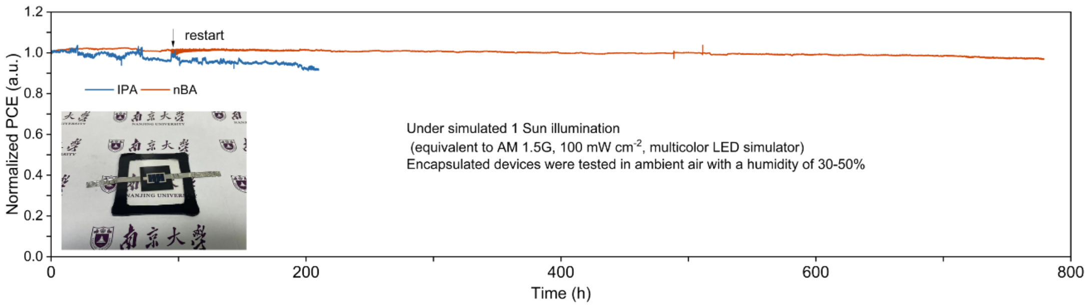

Supplementary Fig. S34 MPP tracking of encapsulated tandem solar cells in air (20–40% relative humidity). Inset is the picture of an encapsulated device.

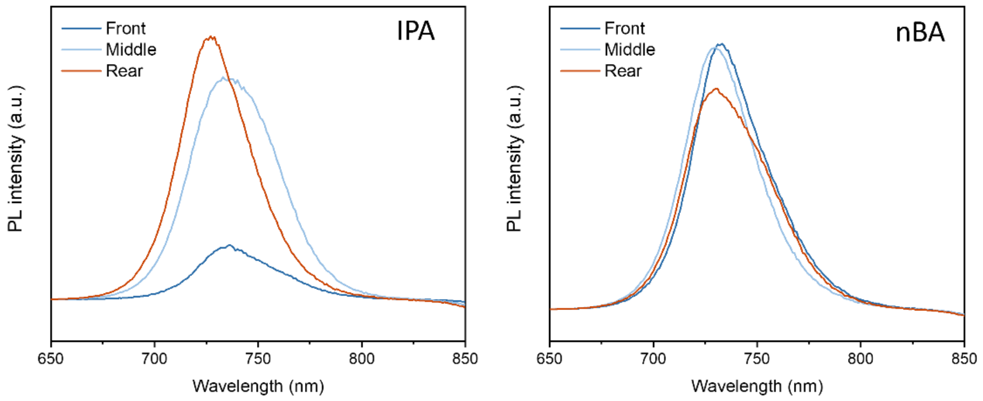

Supplementary Fig. 35 PL spectra of different regions of perovskite films (6 cm x 6 cm) from different regions.

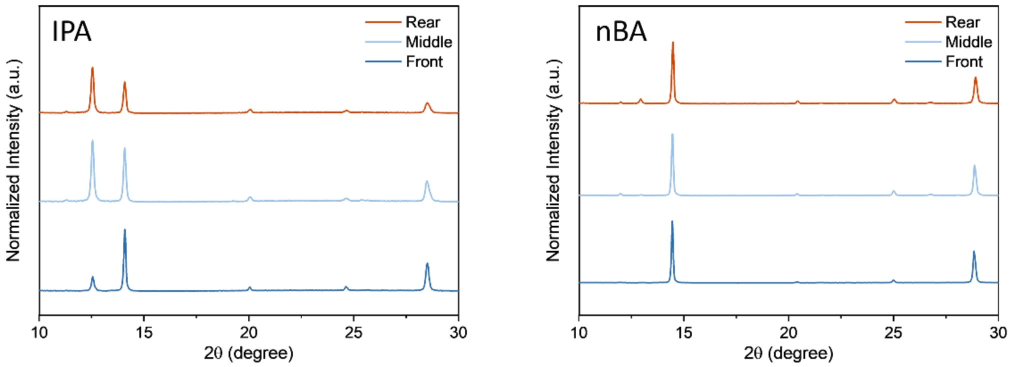

Supplementary Fig. 36 XRD patterns of perovskite films (6 cm x 6 cm) from different regions.

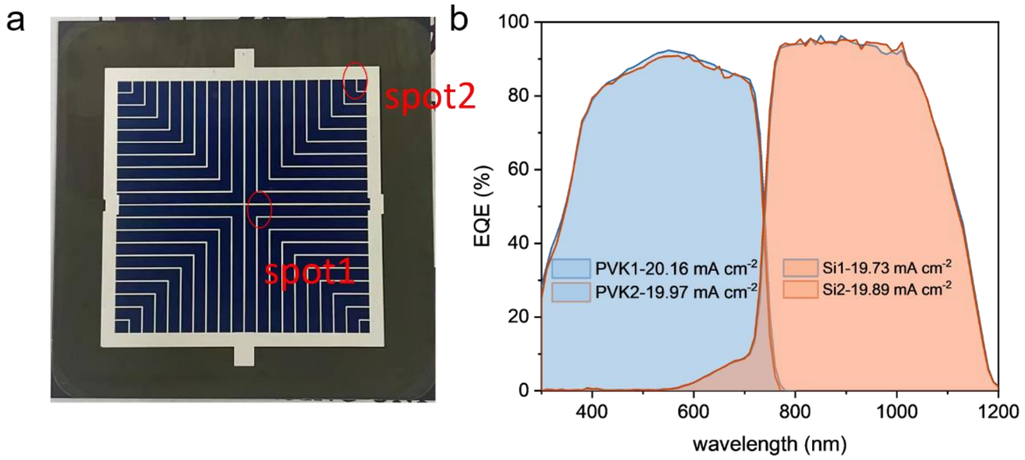

Supplementary Fig. 37 a, Images of perovskite/silicon tandem solar cell. **b**, EQE spectra of the blade-coated textured monolithic perovskite/SHJ tandem cell (16 cm^2) at difference positions.

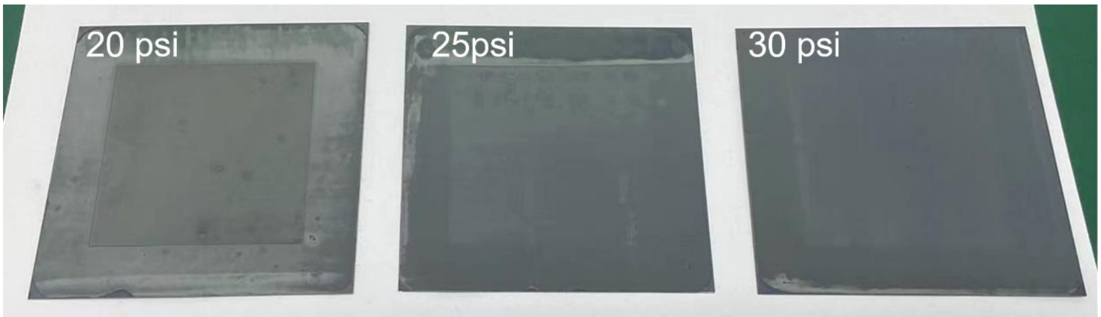

Supplementary Fig. 38 Images of perovskite films fabricated using different quenching gas pressure.

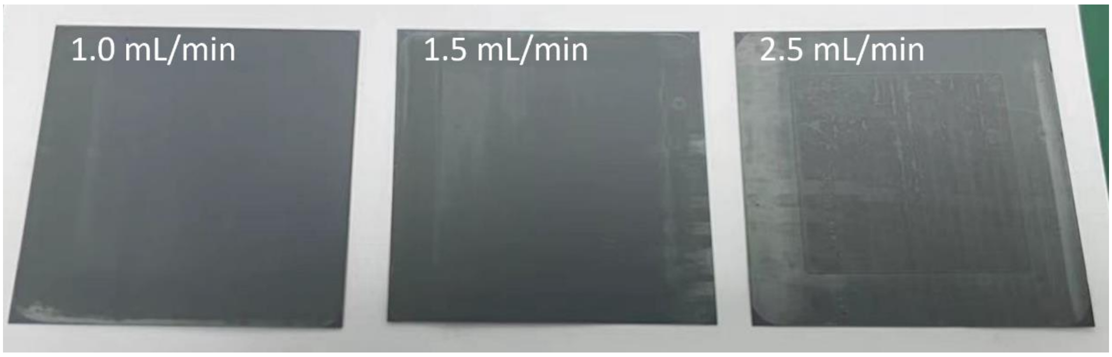

Supplementary Fig. 39 Images of perovskite films fabricated with different injection rate of ink.

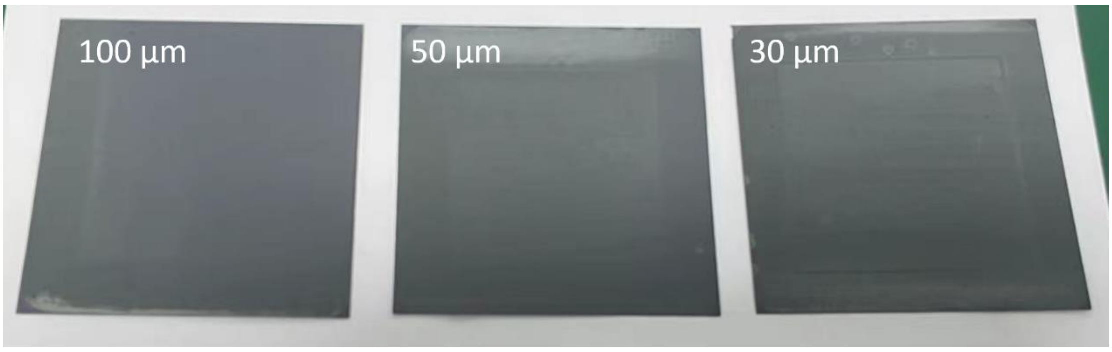

Supplementary Fig. 40 Images of perovskite films fabricated under different gap distances between the blade and substrate.

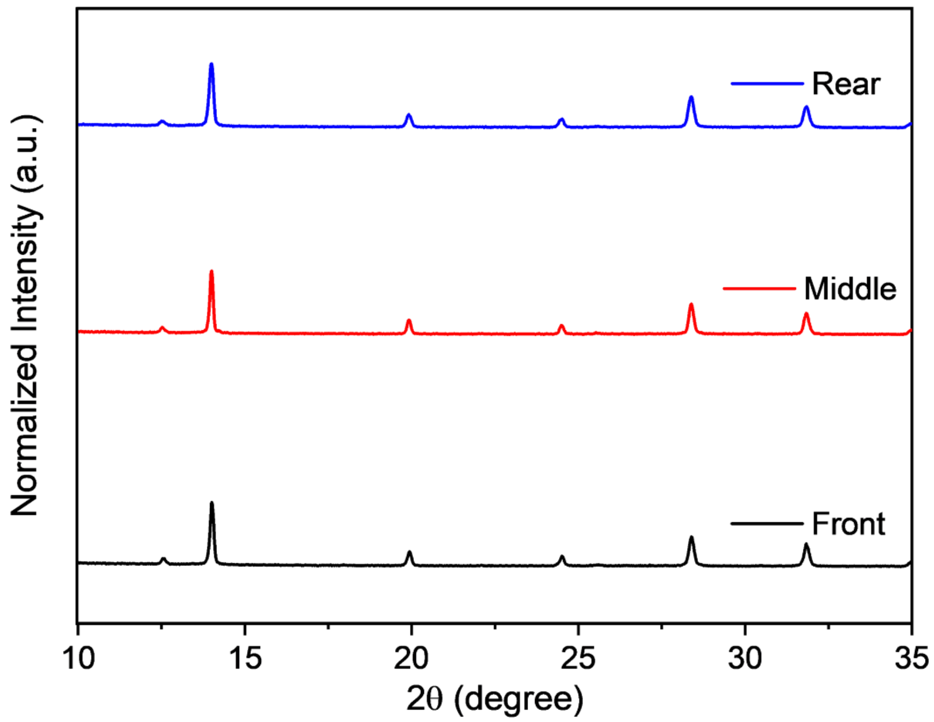

Supplementary Fig. 41 XRD patterns of perovskite films (6 cm * 6 cm) from different regions.

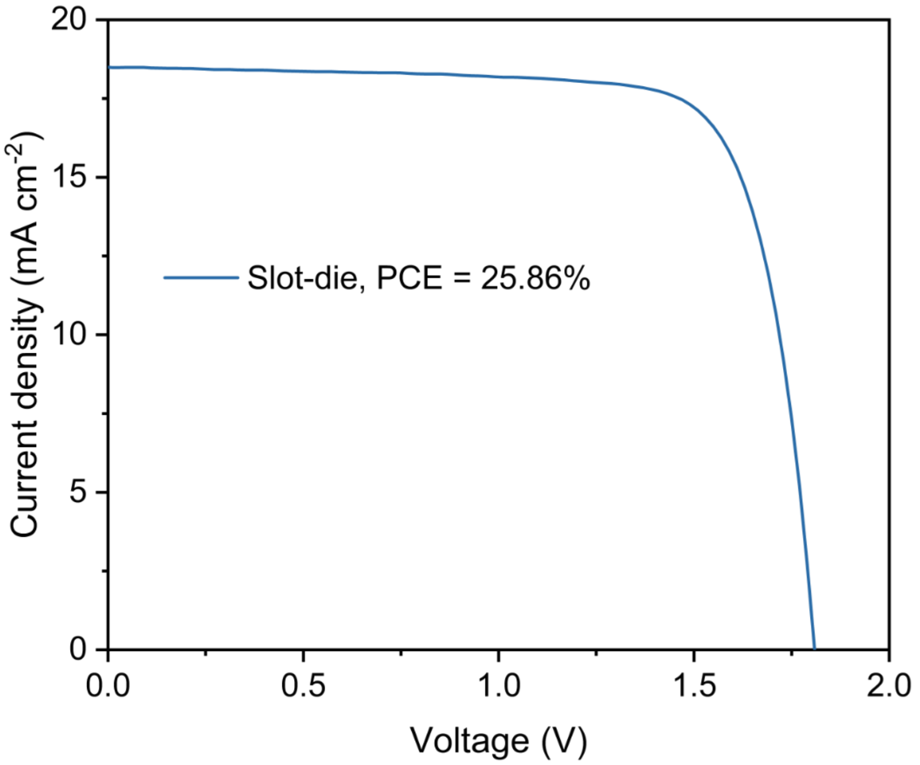

Supplementary Fig. 42 *J*-*V* curve of the perovskite/silicon tandem solar cell ($16\ cm^2$).

Supplementary Table 1. Physical parameters of different alcohols.

**Sample**	**Boiling point (℃)**	**Saturated vapor pressure (kPA) (20℃)**	**Dielectric constant (a.u.)**
EA	78.3	5.8	24.3
IPA	82.5	4.4	19.92
nBA	117.6	0.73	17.8
nPA	137	0.13	13.9

Supplementary Table 2. Extracted TRPL lifetime from Figure 2g using different solvent for perovskite films on glass.

Sample	A_1	τ_1 (ns)	A_2	τ_2	T_{ave} (ns)
EA	191.6	69.5	354.1	172.4	136.3
IPA	774.6	46.6	663.2	263.3	146.6
nBA	833.2	196.9	1038.7	474.2	350.8
nPA	1200.5	78.3	599.6	272.4	142.9

Supplementary Table 3. Average photovoltaic parameters of small area (aperture area 0.049 cm^2) 1.68 eV-bandgap devices with different alcohol.

**Sample**	V_{OC} **(V)**	J_{SC} **(mA cm^{-2})**	**FF (%)**	**PCE (%)**
EA	1.155	18.93	68.20	15.02
IPA	1.194	20.62	78.34	19.30
nBA	1.205	20.88	78.99	19.89
nPA	1.148	20.02	77.67	17.85

Supplementary Table 4. Photovoltaic parameters of champion (0.049 cm^2 aperture area).

Sample	V_{OC} (V)	J_{SC} (mA cm^{-2})	FF (%)	PCE (%)
IPA-R	1.19	20.82	80.98	20.09
IPA-F	1.19	20.80	80.52	19.99
nBA-R	1.22	20.98	81.46	20.77
nBA-F	1.21	21.10	80.89	20.61

Supplementary Table 5. Photovoltaic parameters of champion device (1.044 cm^2 aperture area).

**Sample**	**V_{OC} (V)**	**J_{SC} (mA cm^{-2})**	**FF (%)**	**PCE (%)**
IPA-R	1.20	20.51	74.80	18.40
IPA-F	1.18	20.56	70.26	17.05
nBA-R	1.23	20.85	76.23	19.57
nBA-F	1.22	20.84	75.20	19.14

Supplementary Table 6. Photovoltaic parameters of SHJ with and w/o filter.

**Sample**	V_{OC} **(V)**	J_{SC} **(mA cm^{-2})**	**FF (%)**	**PCE (%)**
W/o filter	0.704	39.48	72.42	20.06
With filter	0.681	16.01	74.58	8.13

Supplementary Table 7. Photovoltaic parameters of champion tandem devices.

**Sample**	**V_{OC} (V)**	**J_{SC} (mA cm^{-2})**	**FF (%)**	**PCE (%)**
IPA-Reverse	1.821	20.01	76.12	27.74
IPA-Forward	1.810	19.95	68.78	24.83
nBA-Reverse	1.828	20.45	78.63	29.39
nBA-Forward	1.814	20.47	75.65	28.08

Supplementary Table 7. Photovoltaic parameters of champion tandem devices (16 cm^2 aperture area).

Sample	V_{OC} (V)	J_{SC} (mA cm^{-2})	FF (%)	PCE (%)
IPA-Reverse	1.807	18.30	74.89	24.75
IPA-Forward	1.783	18.27	65.02	21.18
nBA-Reverse	1.814	18.54	78.33	26.34
nBA-Forward	1.806	18.54	74.80	25.05

Supplementary Table 8. Comparison of reported large-scale perovskite/silicon tandems

**Institution**	**Fabrication Method**	**Aperture area**	***V_{OC}* (V)**	***J_{SC}* (mA cm^{-2})**	**FF (%)**	**PCE (%)**	**Ref**
UNSW	Spin-coating	16 cm^2	1.74	16.2	78	21.9	[2]
NKU	Hybrid two-step	11.9 cm^2	1.85	17.5	70.6	22.8	[3]
NJU	Hybrid two-step	16 cm^2	1.79	18.5	75.7	25.1	[4]
NKU	Hybrid two-step	11.9 cm^2	1.85	18.1	74.9	25.1	[5]
UNC	Blade-coating	24 cm^2	1.89	18.1	73.6	25.2	[6]
NKU	Hybrid two-step	11.3 cm^2	1.87	18.2	74.9	25.4	[7]
NJU	Hybrid two-step	16 cm^2	1.81	18.5	78.3	26.3	This work

Supplementary references

1. Zhang, S. *et al.* The Role of Bulk and Interface Recombination in High-Efficiency Low-Dimensional Perovskite Solar Cells. *Adv. Mater.* **31**, 1901090 (2019).
2. Zheng, J. *et al.* 21.8% Efficient Monolithic Perovskite/Homo-Junction-Silicon Tandem Solar Cell on 16 cm 2. *ACS Energy Lett.* **3**, 2299–2300 (2018).
3. Chen, B. *et al.* A Two-Step Solution-Processed Wide-Bandgap Perovskite for Monolithic Silicon-Based Tandem Solar Cells with >27% Efficiency. *ACS Energy Lett.* **7**, 2771–2780 (2022).
4. Luo, X. *et al.* Efficient Perovskite/Silicon Tandem Solar Cells on Industrially Compatible Textured Silicon. *Adv. Mater.* **35**, 2207883 (2023).
5. Xu, Q. *et al.* Conductive Passivator for Efficient Monolithic Perovskite/Silicon Tandem Solar Cell on Commercially Textured Silicon. *Adv. Energy Mater.* **12**, 2202404 (2022).
6. Yang, G. *et al.* Shunt mitigation toward efficient large-area perovskite-silicon tandem solar cells. *Cell Reports Phys. Sci.* **4**, 101628 (2023).
7. Li, Y. *et al.* CsCl induced efficient fully-textured perovskite/crystalline silicon tandem solar cell. *Nano Energy* **122**, 109285 (2024).
